# A Systematic Review of Obesity Prevention Intervention Studies among Immigrant Populations in the US

**DOI:** 10.1007/s13679-014-0101-3

**Published:** 2014-04-03

**Authors:** Alison Tovar, Andre M. N. Renzaho, Alma D. Guerrero, Noereem Mena, Guadalupe X. Ayala

**Affiliations:** 1 Nutrition and Food Sciences, University of Rhode Island, 112 Ranger Hall, Kingston, RI 02881 USA; 2 Migration, Social Disadvantage, and Health Programs, Global Health and Society Unit, School of Public Health and Preventive Medicine, Monash University; and Centre for International Health, Burnet Institute, Level 3, Burnet Building, 89 Commercial Rd, Melbourne Vic, 3004 Australia; 3 UCLA Center for Healthier Children, Families, and Communities, 10990 Wilshire Boulevard Suite 900, Los Angeles, CA 90024 USA; 4 San Diego State University and the Institute for Behavioral and Community Health, 9245 Sky Park Court, Suite 221, San Diego, CA 92123-4311 USA

**Keywords:** Obesity, Prevention, Intervention, Immigrants, US

## Abstract

The aim of this review was to systematically assess the effectiveness of obesity prevention and control interventions in US immigrant populations across the life course, from preschool-age to adults. A systematic review of relevant studies was undertaken and eligible articles included. The initial search identified 684 potentially relevant articles, of which only 20 articles met the selection criteria, representing 20 unique studies. They were divided into interventions that targeted adults (n=7), interventions that targeted children (n=5) and pilot studies (n=8). The majority of interventions targeted Latinos, predominately Mexican-origin populations. Among the interventions targeting adults, five had an effect on obesity related outcomes. However, they tended to use less rigorous study designs. Among the interventions that targeted children, three had a positive effect on obesity-related outcomes. Three of the eight pilot studies had an effect on obesity-related outcomes. There is a paucity of data on effective interventions but a great need to address obesity prevention to help inform health policies and programs to reduce migration-related obesity inequalities.

## Introduction

Obesity has nearly tripled over the past three decades in the United States (US) [1]. Although obesity impacts all groups, disparities among racial/ethnic groups still exist [1]. Obesity rates among adult non-Hispanic blacks (49.5 %), Mexican Americans (40.4 %), and all other Hispanics (39.1 %) [1] are disproportionately higher compared to non-Hispanic whites (34.3 %) [1]. This pattern is also observed among children and adolescents, with 21.2 % of Hispanic children and adolescents being obese compared to 14.0 % among their non-Hispanic white counterparts [2]. These obesity disparities highlight the need to examine appropriate and effective interventions for diverse populations.

A particularly salient issue among US immigrants is that the risk of becoming overweight or obese increases with greater years living in the US [3–5]. Regardless of country of origin, upon arrival, immigrant adults have a lower prevalence of overweight and obesity than their U.S.-born counterparts. This relative advantage dissipates over time [6], and risk for overweight and obesity increases [3–5, 7–9]. The available evidence suggests an overall positive association between body weight variables and the degree of acculturation (cultural, psychological, and behavioral changes that occur when two or more cultures come into continuous first-hand contact with each other [10]) in both children and adults. In general, these findings suggest that obesity rates are significantly higher among more assimilated immigrants than those who maintain attitudes and behaviors associated with their country of origin [6, 8, 11, 12]. Contributing to changes in weight over time is the increased length of exposure to the US environment, which has been shown to lead to adverse dietary patterns and changes in physical activity [3, 13–20]. These dietary changes are often a function of consuming less traditional foods and consuming more fat, sugar and calories, although this may differ by immigrant group.

With respect to physical activity, longer residence in the US appears to be associated with increased physical activity, although type of physical activity such as occupational and transportation has not been studied [4, 7, 21, 22]. In addition, past research is based on self-reported physical activity, further limiting conclusions that can be drawn. Although exposure to the US environment has been documented as a potential contributor to weight gain among immigrants, the global landscape is changing. Several developing countries are undergoing epidemiologic and nutrition transitions, and some immigrants may no longer be arriving healthy to the US. For example, Mexico is now the leading country of adult obesity [23, 24].

Given the growth of the US immigrant population over the past 40 years [25] and obesity disparities among immigrant populations in general [26, 27], it is critical to identify effective intervention strategies that prevent or control weight gain. To date, published systematic reviews have examined: (1) obesity treatment interventions for Latino adults in the US [28]; (2) overweight and obesity prevention interventions for Latino children [29]; and (3) school-based overweight and obesity prevention interventions for immigrant and non-immigrant student populations [30–34]. Missing from the literature is a systematic review of obesity prevention and control interventions for immigrant populations, in particular, one that includes preschool age children. As such, the aim of this review was to systematically assess the effectiveness of obesity prevention and control interventions in US immigrant populations across the life course.

## Methods

A comprehensive search of the following databases was conducted in October 2013: PubMed; CINAHL with Full Text; and PsycINFO. Additional studies from reference lists of eligible articles and relevant systematic reviews were also identified. The search covered the period from 1995 to 2013.

The following search terms were used by inserting them simultaneously into the databases using MeSH terms and sub-headings: intervention, randomized controlled trial, community based intervention, cluster randomized, quasi experimental, immigrant, migrant, foreign born, Hispanic, Latino, obesity, diet, and physical activity. Terms used within parenthesis (intervention, randomized controlled trial, community based) search terms were divided with ‘or’, while the term groups were connected with ‘and’. We included the search terms “Hispanic” and “Latino” given that research involving this population does not always use the term “immigrant” to describe the sample, and when doing the search without these terms several known articles were not identified. To maximize inclusion of all immigrant groups, we ran the search using terms to describe other immigrant groups (“Asians”, “Middle-Eastern”, “African” and “Indian”), however, no additional articles were found.

The following criteria were used for study inclusion: 1) the intervention targeted an immigrant population in the US, 2) the intervention objective was the prevention or control of obesity, 3) the study examined measured (versus self-reported) outcomes related to obesity, 4) the findings were published in a peer-reviewed journal, and 5) the article was written in English.

**Fig. 1:**
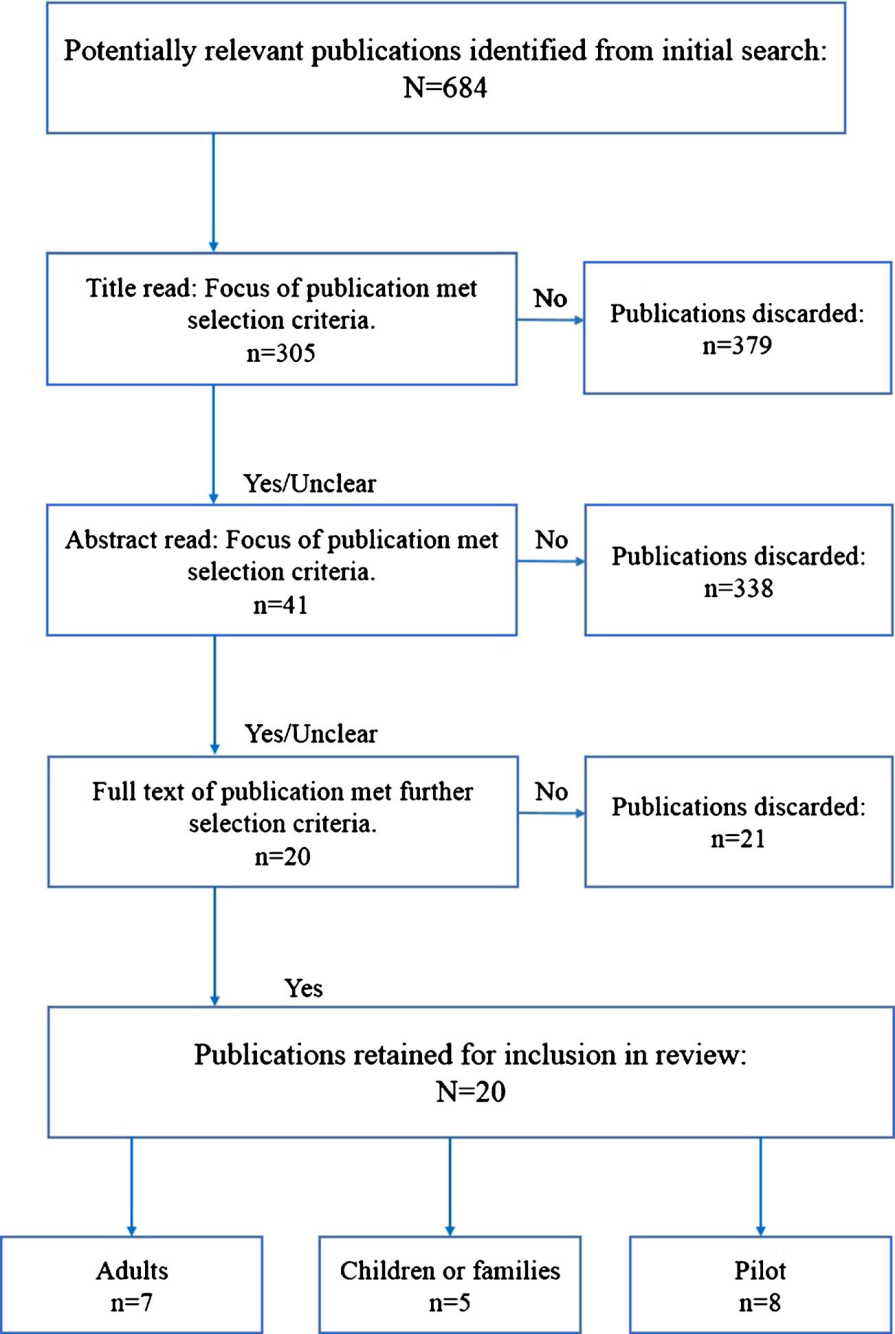
Process of study selection

Following completion of the searches, references were imported into an Endnote library and duplicates identified and removed. The screening process occurred in three steps. In step one, one reviewer screened all initial articles by title (*n* = 684) and excluded those that were not interventions or unrelated to this review’s objectives. In step 2, two reviewers (AT & NM) screened the abstracts of the remaining 305 articles and further excluded articles based on study design (cross-sectional, baseline descriptions), no outcomes reported, a focus on the treatment of obesity and associated chronic conditions, and involving other US-based target populations. In addition, school-based interventions were excluded given recent publications in this area [30–32, 34]. In the last step, the full-text versions of the remaining 41 articles were retrieved and screened for inclusion (Fig. 1). Further exclusions at this stage included obesity treatment studies and those that described methodology and included baseline characteristics only.

Twenty articles met our inclusion criteria, representing 20 unique studies, of which eight were pilot studies. Given the limited research that has been conducted in this area, pilot studies were included despite a potential lack of statistical power to detect an intervention effect. However, they were summarized separately. The included articles were independently reviewed by two co-authors (AT & NM). Then articles were randomly assigned to the rest of the research team (AR, GA, AG) to verify the accuracy of extracted data and to adjudicate any disagreements. Interventions were divided into those that targeted adults only (*n* = 10, including three pilot studies) versus those that targeted children (*n* = 10 including five pilot studies). Two authors (AT & NM) utilized the Quality Assessment Tool for Quantitative Studies as a guide to assess the quality of the studies reviewed [35]. Although the authors did not score the articles, they utilized the topic areas of the tool to guide the description of the selected articles: selection bias, study design, confounders, blinding, data collection methods, withdrawals and dropouts, intervention integrity, and analysis. Details on the cultural adaptation of the intervention strategies and the use of acculturation measures were also noted.

## Definition of Outcomes

Interventions had to have measured obesity-related outcomes: BMI, BMI *z*-score, prevalence of overweight and obesity, percent body fat, waist circumference (WC), and skinfold thickness.

## Results

Twelve studies (seven among adults and five among children) met our inclusion criteria, along with eight pilot studies (three among adults and five among children).

### Interventions Targeting Adults

#### Study and Sample Characteristics

Interventions targeting adults were published on or after 2000 (Table 1). Only two of the seven interventions were randomized controlled trials (Novotny et al. [36] and Cullen et al. [37]). Four used a pretest-posttest design without a control group [38–41], and one was quasi-experimental with a control group [42]. Follow-up periods were relatively short and most commonly ranged from 4 to 12 months, with the longest follow-up period at 24 months. All but one of the interventions was conducted with Latinos, predominantly Mexican origin immigrants. Two interventions either targeted females only or reported findings for females only [38, 40] and in five interventions, at least 50 % of the sample was female [36, 37, 39, 41, 42].

**Table 1:** Description of studies among adults

Author, year	Study design & sample size	Target population & characteristics	Study outcome measures	Attrition rates	Limitations
Elder et al., 2000 [42]	Quasi experimental, n = 732 from17 classes	90 % Latino;51 % female;mean age = 31 yrs.	*Primary outcome*(*s*): BP, WC, HC, and weight.*Secondary*: TC, HDL-C, knowledge, attitudes and diet.	3 months (attrition not reported); 28 % for survey measures and 37 % for physiologic measures at 6 months; no differences by group.	Possible contamination between groups; significant weight gain over time; education-focused vs. behavioral approach, moderately high attrition rate.
Harralson et al., 2007 [40]	Pre-post-test without control, n = 225	100 % Latino;100 % female;mean age = 44 yrs.	*Primary Outcome*(*s*):BMI, BP, WHR.*Secondary*:Health knowledge, depression, psychosocial factors.	48 % at 3 months to 4 months.	No control group, impact of cultural beliefs unclear, short follow-up period, high attrition rate.
Cullen et al., 2009 [37]	Group randomized controlled trial, n = 1107^a^ from 100 classes	89 % Latino;97 % female;mean age = 35 yrs.	*Primary outcome*(*s*):BMI, diet.*Secondary*:Dietary behaviors, psychosocial factors.	26 % at 2 months and 45 % at 4 months; no differences by group at follow-up (data not presented).	Short follow-up period, high attrition rate at 4 months.
Ayala et al., 2011 [38]	Pre-post-test without control n = 387^b^	100 % Latino;100 % female median age = 39 yrs.	*Primary outcomes*(*s*):BMI, PA. *Secondary*:WC, BP, fitness, flexibility, depression, psychosocial factors.	47 % at 6 months and 39 % at 12 months.	No control group, PA focus only, high attrition rate at 6 months.
Novotny et al., 2012 [36]	Cluster randomized controlled trial, n = 4263^c^ from 30 hotels	42 % Filipino, 32 % Other Asian, 13 % Pacific Islander, 9 % White, 1 % African-American, 52 % female;mean age = 46 yrs.	*Primary outcome*(*s*): BMI, Waist-to-height ratio.*Secondary*: PA and diet.	17 % at 12 months and 30 % at 24 months; Rates by group not reported.	Unclear if control and intervention groups were similar at beginning of study, moderately high attrition rate at 24 months.
Ziebarth et al, 2012 [41]	Pre-post-test without control, n = 47 families (57 adults and 54 children)^d^	100 % Latinos; 89 % female; mean age = 32 yrs.	*Primary outcome*(*s*):BMI, WC, BP, blood lipids, glucose	0 % at 2 months.	No control group, short follow-up period, small sample size.
Schwartz et al., 2013 [39]	Pre-post-test without control, n = 477^e^	“vast majority Latino”(% not reported);61 % female;mean age = 40 yrs.	*Primary outcome*(*s*): BMI, WC, HC, BP, blood lipids, glucose, A1C *Secondary*: PA	2 % at 2 months and 35 % at 12 months	No control group, moderately high attrition rate at 12 months.

A1C = glycated hemoglobin, BMI = body mass index, BP = blood pressure, HC = hip circumference, HDL-C = high-density lipoprotein cholesterol, PA = physical activity, TC = total cholesterol, WC = waist circumference, WHR = waist-to-hip ratio

^a^Analyses excluded 101 participants who consented but were pregnant.

^b^Analyses excluded 34 women over 60 years of age given different evaluation protocols used and 16 males given the limited generalizability of this subsample.

^c^Analyses excluded 2624 participants who were not overweight or obese immigrants.

^d^No biometric data were collected from children.

^e^Analyses excluded 114 participants who had not yet completed the intervention

#### Summary of Interventions

Six of the seven interventions targeting adults were conducted in community settings, Schwartz et al. [39] included an additional home visit component and Novotny et al.’s study [36] was conducted in a work environment (hotels) (Table 2). Interventions ranged in length from 2 to 12 months. Ayala et al. [38] and Schwartz et al. [39] implemented interventions delivered by trained promotores, Elder et al. [42] and Cullen et al. [37] had interventions delivered by staff from existing community programs (ESL and EFNEP) and the remaining were conducted by trained bi-lingual staff [36, 41] with the exception of the report by Harralson et al. [40] which did not specify who delivered the intervention. The majority of the interventions targeted both diet and physical activity using educational and other health promotion strategies. Two of the studies had a concurrent focus on improving children‘s health behaviors and therefore included additional components related to family health [37, 41].

**Table 2:** Summary of interventions by setting and number of intervention components among adults

Author, year	Setting	Delivery mode	No. of intervention components*	Planned dose and fidelity	Intervention effects	Major findings
Elder et al., 2000 [42]	ESL classes	ESL teachers	1 component: *Intervention*: Nutrition/heart health education integrated into ESL classes. *Control*: Attention control on stress management.	Dose: Up to five 3-hr classes over a 1-to 2-week period over 3 months. Fidelity: ESL teachers received a half-day training however fidelity not reported.	X	Increase in HDL (p < 0.01) at 3 months vs. control, changes not maintained at 6 months. TC and BP decreased in both groups (p < 0.001). Increase in weight over time for both groups (p < 0.001).
Harralson et al., 2007 [40]	Women’s wellness center	Not mentioned	2 components: *Intervention*: PA classes and health education sessions. *No control group*.	Dose: Three 1-hour PA classes per week. One 30-minute health education class per week. First year: 12 weekly sessions. Second year: 16 weekly sessions. Fidelity: Not reported.	√	39 % of women BMI > 30 compared to 50 % at baseline. Decrease in BMI (p = 0.001), abdominal obesity (p < 0.001), and WHR (p = 0.04).
Cullen et al., 2009 [37]	Texas EFNEP classes	6 bilingual paraprofessionals plus separate EFNEP staff members for control group	4 components: *Intervention*: traditional EFNEP classes plus six videos, weekly goal setting and review, and intervention materials including recipes. *Control*: traditional EFNEP classes.	Dose: 6 EFNEP class sessions. Fidelity: Selected paraprofessionals attended a 2-day training prior to intervention. 46 intervention sessions in 29 classes were observed, fidelity >80 %.	√	Significant decrease in BMI in the intervention group at 2 months (p < 0.05), changes not maintained at 4 months.
Ayala et al., 2011 [38]	Various community settings (i.e., schools, recreation centers, health centers)	Bilingual (Spanish and English) or monolingual Spanish speaking trained promotores	1 component: *Intervention*: group exercise classes delivered throughout the community. *No control group*.	Dose: Mean attendance was 16 sessions per group exercise sign-in sheets. Fidelity: Promotores received an 8-session training, however fidelity not reported.	√	No significant changes in BMI. Improved systolic BP, WC, and fitness indicators at 12 months (all p < 0.001).
Novotny et al., 2012 [36]	Hotels	Research staff	3 to 4 components: *Intervention*: Hotel environmental changes (i.e., electronic sign messages, highlighting of healthy cafeteria and menu foods). Weekly group-based nutrition and PA classes held at work for all employees. Weekly group-based nutrition and PA outside of work hours for obese individuals only. Feedback on weight and WC measures. *Control*: Only feedback on weight and WC measures.	Dose: Assessment and feedback on physical measurements and health behaviors at baseline, 12-, and 24-months (Up to three possible assessment and feedback sessions over 24-month period). Intervention group attended weekly 30 min sessions that incorporated PA and nutrition education for 24 months. Content of sessions was reinforced daily by the changes to the hotel environment. Fidelity: Not reported	X	No significant changes in BMI and WHR.
Ziebarth et al., 2012 [41]	Community health center	Bilingual staff and certified bilingual exercise instructor	3 components: *Intervention*: We Can! curriculum (parents and children separately), PA sessions (together), and a family dinner. *No control group*.	Dose: Eight weekly classroom (40 mins) and PA sessions (40 mins), followed by a family dinner. Education sessions conducted by bilingual health educator and registered nurse. PA sessions conducted by bilingual health promoter/certified exercise instructor. Fidelity: Not reported.	√	Decrease in weight (p = 0.01), BMI (p = 0.01), reduction in sBP and dBP (p = 0.01, p = 0.05), decrease in blood glucose (p = 0.03) at 2 months.
Schwartz et al., 2013 [39]	Community setting and home visits	Bilingual promotores	4 components: *Intervention*: Weekly group sessions that included a general family activity, PA, healthy meal or snack, discussion and wrap-up. Weekly family home visit. Review of baseline and post-assessment labs with clinicians. *No control group*.	Dose: Eight two-hour group sessions and eight one-hour home visits over a 3 month period. Promotores selected from target communities were trained to conduct group-sessions and home visits. Fidelity: Promotores were trained to conduct family-meetings and home visits, however fidelity not reported.	√	Significant reductions in all primary outcomes from baseline to 2 months (p < 0.000), and remained significant at 12 months (Weight, p = 0.001, BMI, WC, HC, WHR (p < 0.000), except for BP and blood lipids.

X = no effect on obesity related outcomes; √= had an effect on obesity related outcomes

BMI = body mass index, BP = blood pressure, sBP = systolic blood pressure, dBP = diastolic blood pressure, EFNEP = Expanded Food and Nutrition Education Program, ESL = English-as-a-Second-Language, FU = follow-up, HC = hip circumference, HDL-C = high-density lipoprotein cholesterol, PA = physical activity, TC = total cholesterol, WC = waist circumference, WHR = waist-to-hip ratio

*Intervention components refers to interventions strategies employed

#### Intervention Effects

Five of the seven interventions had an effect on obesity-related outcomes [37–41]. Those that found an effect tended to be non-experimental and quasi-experimental interventions. Among the two randomized controlled trials, only Cullen et al. [37] found a significant decrease in BMI at immediate post intervention (2 months), although this was not maintained at the 4-month follow-up. In this study, 100 Expanded Food and Nutrition Programs (EFNEP) (*n* = 1107) were randomized to receive a modified curriculum to promote healthy home food environments and parenting skills related to obesity prevention over 6 weekly sessions to children’s parents; the control condition received traditional Texas EFNEP classes (Table 2). Intervention fidelity was >80 % and was assessed by observation of 46 intervention sessions. Although much of the focus of the intervention was geared toward improving the family’s eating, only the parents participating in the intervention were assessed for anthropometrics and household information. This intervention was designed within an already existing program, thus maximizing the potential for sustainability. However, there was a high attrition rate (45 % at 4 months). The other randomized controlled trial involved a multi-center cluster randomized controlled trial that included 30 hotels located in Oahu, HI [36]. Nearly half of the employees were Filipino (42 %), while the remaining identified as other Asian (32 %), Pacific Islander (13 %), White (9 %), and African-American (1 %). Intervention hotel employees received a group-based intervention designed to raise awareness of their weight, health habits and health relevant features of the work environment. However, no significant changes were seen in BMI at the 24-month follow-up. No information was provided on intervention fidelity.

Of the five studies using a less rigorous study design, three reported significant improvements in BMI [39–41], one reported significant improvements in WC at 12 months [38], and another showed improvements in intermediate outcomes such as high-density lipoprotein cholesterol (HDL-C), total cholesterol (TC), and blood pressure (BP) at 3 months, but no improvements in primary obesity-related outcomes [42]. The quasi-experimental studies demonstrating improvements in obesity-related outcomes were community-based and engaged the community of interest to some degree [40, 41], although some used more participatory approaches than others [38, 39]. For example, Schwartz et al. [39] worked with the Idaho Partnership for Hispanic Health to design and conduct a 3-month intervention (including eight two-hour group sessions and eight one-hour home visits) ) which targeted both healthy eating and physical activity, as well as provided information on health conditions. The home visits involved the entire family. This intervention not only found significant reductions in weight, BMI, WC, and BP over 2 months in a sample of 363 adults, but also significant reductions in TC, low-density lipoprotein cholesterol (LDL-C), glucose and hemoglobin A1C. Ayala et al. [38] worked with several community-based organizations, through a funded academic-community partnership, to design and conduct a physical activity intervention, “Familias Sanas y Activas” led by promotores. Promotores led between 2-4 exercise classes per week and participant measures were conducted at baseline, and 6 and 12 months post-baseline. They found significant improvements in WC, a primary outcome, in addition to improvements in systolic BP, fitness and hamstring flexibility, among other changes.

Harralson et al. [40], in their implementation of “Un Corazon Saludable”, also engaged with community members to develop their intervention, although few details are provided on this process. The intervention consisted of an exercise program plus nutrition education modules (three 1-hour PA classes per week and one 30-minute health education class per week). In the first year, there were 12 weekly sessions; this was increased to 16 weekly sessions in the second year given participant demand. Of those women completing the intervention, the number of women with a BMI ≥ 30 kg/m2, dropped from 50 % at baseline to 39 % at the end of the program. A significant decrease in BMI, abdominal obesity, and waist-hip-ratio (WHR) were observed. Through their community partnerships, Ziebarth et al.[41] also showed significant improvements in BMI and other measured outcomes following implementation of a 2-month program which promoted awareness of healthy food choices during weekly classes led by a bilingual health educator and a nurse, and provided an opportunity to do physical activity led by a certified exercise instructor.

It is worth noting that Elder et al. [42] designed an intervention integrated into an existing program for immigrants. The intervention group received nutrition and heart health education classes integrated into their English as a Second Language classes (up to five 3-hour classes) compared to the control group that received classes on stress management over a 3-month period. Compared to the control, significant increases in HDL were observed in the intervention group. However this change was not maintained at the 6-month follow-up. A significant increase in weight occurred over time in both groups.

### Interventions Targeting Children

#### Study and Sample Characteristics

Interventions targeting children were published after 2005 (Table 3). Of the five studies, four were randomized controlled trials and one used a quasi-experimental design. All of the interventions among children (*n* = 3) and families (*n* = 2) were conducted with Latino populations (at least 50 % of the target population), the majority being Mexican origin. Only one intervention collected follow-up data beyond 12 months.

**Table 3:** Study characteristics for interventions among children

Author, year	Study-design and sample size	Target population & characteristics	Study outcome measures	Attrition rates	Limitations
Fitzgibbon et al., 2006 [45]	Group randomized controlled trial, n = 401 children from 12 Head Starts	81 % Latino; 49 % female; mean age = 4.2 yrs.	*Primary outcome*(*s*): BMI. *Secondary*: dietary intake, and PA.	3 % for intervention and control at 3.5 months, 11.9 % for intervention and 17 % for control at 12 months and 12.9 % for intervention and 17 % for control at 24 months.	Small sample size, lack of community engagement which could have enhanced cultural appropriateness of intervention, minimal parental engagement.
Barkin et al., 2012 [46]	Parallel-group randomized controlled trial, n = 106^a^ parent child-dyads	95 % Latino; Parent: mean age = 31.3 yrs. Child: 50 % female; mean age = 4.2 yrs.	*Primary outcome*(*s*): BMI, BMI percentile.	35 % for intervention and 23 % for control at 3 months.	Short follow up period, small sample size, moderately high attrition in intervention versus control group.
Yin et al., 2012 [44]	Pre-post with two treatment groups and a comparison n = 423^b^ children from 4 Head Starts	90 % Latino; 52 % female; mean age = 4.2 yrs.	*Primary outcome*(*s*): BMI-z score.	Unknown by intervention and control; overall 12 % at 4.5 months.	Short follow up period, non-randomized, Not intending to stratify analyses by center & home based.
Bellows et al, 2013 [43]	Group randomized controlled trial, n = 274^c^ children from 8 Head Starts	59 % Latino; 45 % female; mean age = 4.4 yrs.	*Primary outcome*(*s*): PA *Secondary*: BMI, BMI percentile and BMI-z score, fine and gross motor skills.	26 % for intervention and 21 % for control at 4.5 months.	Short follow up period, no data on parental/household characteristics, dose of intervention may not have been adequate, no dose-response analysis; questionable use of nutrition education as control.
Haines et al., 2013 [47]	Randomized controlled trial, n = 121^d^ families	51 % Latino; Child: 52 % female, mean age = 4.1 yrs.	*Primary outcome*(*s*): change in eating meals together as a family, child's sleep duration, child's TV viewing time, and presence of TV in the room where child slept. *Secondary*: child BMI.	11.3 % for intervention and 5.1 % for control at 6 months.	Although formative work with population, no reported community engagement.

BMI = body mass index, BMI-z = body mass index z-score, PA = physical activity, TV = television

^a^Analyses excluded 31 participants without post-test data.

^b^Analyses excluded 39 participants without post-test data.

^c^Analyses excluded 73 participants who were lost to follow-up.

^d^Analyses excluded 10 participants who were lost to follow-up

#### Summary of Interventions

Interventions targeting children were conducted in a number of settings, including Head Start programs [43–45], community recreation centers [46], or home-based [47] (Table 4). Interventions ranged in length from 3 to 6 months. The pre-school interventions were delivered by trained teachers/early childhood educators, while the others were delivered by trained bi-lingual workers. The pre-school interventions had at least two intervention components related to nutrition and physical activity; the home and community based interventions included additional components related to building social networks [46], and improving family routines through text-messaging [47].

**Table 4:** Summary of interventions by setting and number of intervention components among children

Author, year	Setting	Delivery mode	No. of intervention components*	Planned dose and fidelity	Intervention effect	Major findings
Fitzgibbon et al., 2006 [45]	Pre-schools through the Archdiocese of Chicago	Delivered to children by trained early childhood educators in both Spanish and English	2 components delivered to children (with a parent component including newsletters): *Intervention*: PA (20 min of aerobic activity) + Nutrition education (20 min of nutrition activity based on hand puppets reflecting food pyramid). *Control*: General health.	Dose: *Intervention*: 3 days/week for 3.5 months plus 12 weekly parent newsletters/homework assignments; *Control*: weekly 20 min sessions for 3.5 months. Fidelity: not reported.	X	No significant BMI differences between intervention and control schools.
Barkin et al., 2012 [46]	Public community recreation center	Delivered by one trained facilitator in Spanish	4 components delivered to parents: *Intervention*: Group skill building sessions around nutrition, PA, sedentary behaviors, and building social networks. *Control*: school readiness activities.	Dose: *Intervention*: Weekly 90 min. sessions for 3 months; *Control*: Three 60 min. sessions over 3 months. Fidelity: Facilitator training and supervision; verification of essential treatment components by supervisor; controlling for differences between interventionists by having same administer each condition; collection of fidelity measures (e.g., length, #, frequency of sessions; participation rates). A study team member observed 3 sessions/condition (100 % of key messages discussed).	√	Controlling for covariates, effect of treatment on post-intervention BMI was significant. Intervention effect strongest on obese children.
Yin et al., 2012 [44]	Pre-school with parent involvement at school	Delivered by trained teachers in the pre-school centers, by peer-educators to parents	3 components delivered to children, centre staff, and parents. *Intervention*: PA (gross motor program + outdoor play), nutrition (healthy eating using Sesame Street characters), health literacy and capacity building *Control*: Delayed intervention.	Dose: Center-based intervention: different modules, 2 weeks each, teachers needed to use all activities for each module at least once during 2 wks, integration of activity into daily routines (free play, healthy eating promotion). Home-based intervention: Center-based activities + 6 poster information sessions led by peer parent educators for 4.5 months (18 wks); *Control*: Received intervention materials and training upon completion of study. Fidelity: levels of adherence to the protocol and its implementation, intervention exposure and program participation (e.g. teachers completed a biweekly report on supplemental classroom activities (frequency, time of the day, location of use, and problems) although results on fidelity measures not reported.	√	Weight gain in weight z score for age and gender was significantly less in intervention (center + home based) vs. control but not just for center based.
Bellows et al., 2013 [43]	Pre-schools (i.e. Classrooms)	Delivered by trained teachers in English	2 components delivered to children: *Intervention*: PA (focused on group of skills from one of the three gross motor skill categories) + nutrition (Food Friends program to increase willingness to try new foods). *Control*: nutrition only.	Dose: *Intervention*: 4 days/wk. for 15-20 min in classrooms for 4.5 months (18 wks); *Control*:12 week nutrition program Fidelity: Teachers trained on the study protocol before the study; program surveys for teachers every 3 weeks to ascertain activity completion; fidelity to lessons measured with Likert scale, although results of fidelity measures not reported.	X	Intervention had no effects on weight status.
Haines et al., 2013 [47]	Home- based	Delivered by trained bilingual workers	3 components delivered to parents *Intervention*: Coaching, mail-based education, and text-based prompt targeting family functioning, nutrition and meal patterns, and PA and sedentary behaviours. *Control*: Focused on healthful development.	Dose: *Intervention*: 4 home visits, 4 health coaching calls, mailed materials, twice weekly text messages for 4 months and then weekly for 2 months; *Control*: 4 monthly mailings Fidelity: Training of health educators on motivational interviewing; monthly coaching to reinforce messages, kept detailed records of completed home visits and calls although results of fidelity measures not reported.	√	Intervention participants had significant decreases in BMI.

X = no effect on obesity related outcomes; √= had an effect on obesity related outcomes

BMI = body mass index, PA = physical activity

* Intervention components refers to interventions strategies employed

#### Intervention Effects

Three of the five interventions, two randomized controlled trials [46, 47], and one quasi-experimental [44] had a positive effect on obesity-related outcomes (Table 3). Among the randomized controlled trials, Haines et al. [47] and Barkin [46] et al. reported significant improvements in BMI outcomes, one led to significant improvements in gross motor skills but not in BMI [43], and one found no differences in obesity-related outcomes [45].

The three studies that showed improvements in BMI targeted the primary caregiver in order to influence the child’s health behaviors. Haines et al.[47] completed a 6-month intervention with 121 families, which included four home visits, four coaching calls, educational materials, and weekly text messages (Table 4). The intervention materials focused on promoting four household routines (family meals, adequate sleep, limiting TV time, and removing the TV from the children’s bedroom). Health educators, who delivered the intervention, were trained on motivational interviewing techniques and detailed records of completed home visits were kept to assess fidelity; however, results of fidelity measures were not reported. Similarly, Barkin et al. [46] also involved parents (*n* = 106 dyads) through 12 weekly 90-minute skill building sessions, at community recreation centers, designed to improve family nutritional habits and increase physical activity. Although the intervention was successful, the attrition rate for the intervention group (35 %) was higher compared to 23 % for the control at 3 months. Intervention fidelity data were collected such as length and frequency of sessions, and observations were conducted of the sessions; 100 % of key messages were discussed. Both of these interventions were relatively short; sustainability of these interventions remains unclear.

The one quasi-experimental study, which was pre-school/child care center-based, showed favorable changes in sex-specific weight-for-age z scores among children who received an in-center plus home-based intervention [44]. In this study, there were two treatment centers (one received a center based intervention and another received both the center based and a home-based intervention) and one comparison center (*n* = 423). The home-based intervention involved a novel approach of reaching parents through peer-led education during child care pick up times. Parents were invited to view informational posters and discuss healthy eating and physical activity strategies with the peer educators.

The two randomized controlled trials that did not see intervention effects on BMI were both pre-school/child care center-based. Bellows et al. [43] worked with eight Head Start centers to conduct an intervention, which consisted of 15-20 min lessons, 4 days a week for 4.5 months (Table 4). The lessons centered primarily on improving gross motor skills but also incorporated a nutrition program for both the intervention and control classrooms [43]. Fidelity to lessons was assessed using a 5-point Likert scale on surveys administered to teachers, although results of fidelity measures were not reported. Unfortunately, no parental or household data were collected and they did not control for Head Start site. It is also unclear how having a control group that received a nutrition education program might influence the outcomes of interest. Hip-Hop Health Jr. for Latino preschool children targeted 12 Head Start centers (*n* = 401) to participate in a group randomized controlled trial whereby intervention centers received a diet/physical activity curriculum for 3.5 months (three times a week) with follow-up data collection periods at 12 and 14 months post baseline measurements [45]. Target behaviors included increasing fruit and vegetable consumption and physical activity, and decreasing fat intake and sedentary behavior. Although parents were engaged through weekly newsletters and homework assignments, their engagement was minimal. In addition, there was no evidence of community engagement to inform the cultural appropriateness of the intervention.

### Pilot Studies

Among the pilot studies, three targeted adults [48–50] and five targeted children [51–55]. All of the adult interventions were quasi-experimental and two of the interventions targeting children were randomized controlled trials (Table 5) [51, 55]. All of the pilot studies were completed with a predominantly Latino population and one included a Somali and Cambodian population [50]. Among the adult studies, one study showed positive effects on BMI [48] and one showed significant improvements in BMI at 9 months [49]. Among children, two showed significant improvements in BMI: one among children with a BMI percentile > 50 [51] and one among a subset of children who were overweight and obese [52] (Table 6).

**Table 5:** Description of pilot studies among adults and children

Author, year	Study design & sample size	Target population & characteristics	Study outcome measures	Attrition rates	Limitations
Adult interventions					
Keller and Cantue, 2008 [49]	Pre-post-test clinical trial, n = 18	100 % Latino; 100 % female; mean age = 55 yrs.	*Primary outcomes*(*s*): Blood lipids, % and location of BF, BP, and adherence to PA.	Group I: 64 % Group II: 43 % at 9 months.	No control group, high attrition rate at 9 months.
Millard et al., 2011 [48]	Pre-post-test with control, n = 91^a^	89 % Latino; 98 % female; mean age = 36 yrs.	*Primary outcome*(*s*): BMI.	21 % at 2 months; Rates by group not reported.	Short follow-up period, excluded 20 % of participants who attended less than half of sessions from analysis.
Wieland et al., 2012 [50]	Pre-post-test without control, n = 45^b^	44 % Latina, 31 % Somalia, 18.7 % Cambodian, 6 % African-American; 100 % female; mean age = 40 yrs.	*Primary outcome*(*s*): Weight, BMI, WC, and BP. *Secondary*: QOL.	29 % at 1.5 months.	No control group, short follow-up period.
Child interventions					
Olvera et al., 2010 [54]	Two-arm parallel group assignment; n = 46 mother-daughter dyads	100 % Latino; 100 % female; Mother: mean age 35.8 yrs.; Daughter: mean age = 10.2 yrs.	*Primary outcome*(*s*): Physical fitness and activity. *Secondary*: Diet, BMI.	31 % of dyads in intervention group versus 15 % of dyads in control group at 3 months.	No randomization, short follow-up period, moderately high attrition rate in the intervention group.
Slusser et al., 2012 [51]	Randomized controlled trial; n = 160 families ^c^	100 % Latino; Mothers: 100 % female mean age 31.6 yrs. Child: 56 % female	*Primary outcome*(*s*): BMI.	*28 % in intervention group and 38 % in control group at 12 months.	Moderately high attrition in control group, sub-set analysis completed of those BMI >50 percentile is unclear, Time 2 for intervention not discussed, no community involvement reported.
Castro et al., 2013 [52]	Pre-post-test without control; n = 120 children, 60 families	Child: 59 % Latino; 51 % female; mean age = 6 yrs.	*Primary outcome*(*s*): BMI. *Secondary*: Access to fruits and vegetables.	20.8 % at 1.8 months (7 wks).	No control group, short follow-up period, non-independent observations.
Bender et. al., 2013 [53]	Pre-post-test without control; n = 33 mother-child dyads	100 % Latino dyads; Mothers: mean age = 27.0 yrs., Child: 52 % female; mean age = 3.6 yrs.	*Primary outcome*(*s*): Children’s sugar sweetened beverage consumption and maternal walking. *Secondary*: BMI.	9 % at 6 months.	No control or randomization.
Fitzgibbon et al., 2013 [55]	Randomized controlled trial; n = 146 children, 123 parents	94 % Latino; Parent: 89 % female; mean age = 32.8 Child: 50 % female; mean age = 4.5 yrs.	*Primary outcome*(*s*): BMI. *Secondary*: PA, diet, screen time.	1 % at 5.3 months (14 wks) and 15 % at 12 months for intervention and 3 % 5.3 months (14 wks) and 9 % at 12 months for control.	Homogenous group of low-acculturated and low-income Latinos.

BMI = body mass index, BP = blood pressure, CBPR = Community Based Participatory Research, PA = physical activity, QOL = quality of life, WC = waist circumference, *attrition rates based on sample size after exclusion at baseline for BMI < 50th percentile

^a^Analyses excluded 10 cases with insufficient attendance data.

^b^Analyses excluded 13 cases with no follow-up data.

^c^Analyses excluded 39 children with a BMI <50th percentile

**Table 6:** Summary of pilot interventions by setting and number of intervention components among adults and children

Author, year	Setting	Delivery mode	No. of intervention components	Planned dose and fidelity	Intervention effect	Major findings
Adult interventions						
Keller and Cantue, 2008 [49]	*Barrios* (neighborhoods) and community centers	Promotoras	1 component:Both treatment groups: PA intervention, different frequencies of walking.	Dose: *Group I*: 30 min for 3 days/wk of walking*Group 2*: 30 min for 5 days/wk of walking. Each participant walked at a 3.2-MET intensity for 9 months. Fidelity: Not reported	√	BMI decreased over time for both groups; it decreased significantly at 9 months for group I but not for group II.
Millard et al., 2011 [48]	*Colonias* (social networks in low-income areas)	Promotores	2 components: *Intervention*: PA (aerobics and stretching), chronic disease education. *Control*: No intervention.	Dose: Weekly group meetings (20 min of PA) over 1.8 months (7 wks) Fidelity: Training of Promotores but fidelity not reported.	√	Significant decrease in BMI.
Wieland et al. 2012 [50]	Different community settings (YMCA) worked with Rochester Healthy Community Partnership	Research staff	2 components:Nutrition education (healthy food choices, portion sizes) and PA (dance and strength training).	Dose: Two 90 min classes (60 min PA and 30 min nutrition education) provided weekly for 1.5 months (6 wks) Fidelity: Not reported	X	Decreasing trend in biometric data but not statistically significant.
Child Interventions						
Olvera et al., 2010 [54]	Various community settings (i.e. community centers, parks, grocery stores) and school setting (i.e. classroom, gym, playground, cafeteria)	Delivered by instructor	3 components delivered to mothers and daughters: *Intervention*: Nutrition education, PA and behavioral counseling.*Control*: Educational material on nutrition and counseling plus light intensity PA. *Control*: Weekly meetings with instructor for 45 min + 45 min of light intensity aerobic or sports session.	Dose: *Intervention*: Over 3 months: 3 weekly structured group aerobic classes, 2 weekly nutrition sessions, and 1 weekly behavioral counseling session were provided. Each session included 45 min of exercise and 45 min of either nutrition education or counseling. Fidelity: Not reported.	X	No significant BMI differences between mothers and daughters of intervention and control.
Slusser et al., 2012 [51]	Health clinics and Head Start programs and preschools	Trained bilingual social worker	3 components delivered to mothers: *Intervention*: Parenting, nutrition (“Bright Futures in Practice Nutrition”) and physical activity (“Bright Futures in Practice PA”) *Control*: Wait list control (offered classes 1 yr. post).	Dose: Seven weekly 90-minute sessions plus two boosters delivered over 4 months. Fidelity: Not reported.	√	Children in intervention decreased BMI-z scores significantly vs. controls at 12 months (among subset of children >50th percentile).
Castro et al., 2013 [52]	Community gardens	Delivered by cooperative extension and research staff	3 components delivered to families:*Intervention*: Gardening, cooking and nutrition, social events. *No control group*.	Dose: Weekly garden and cooking and nutrition sessions; 4 social events over 1.7 months (7 wks). Fidelity: Not reported.	√	Of overweight and obese children (38 %), 17 %, achieved statistically significant improvements in BMI classification.
Bender et. al., 2013 [53]	Urban Health Center	Delivered by trained Promotora	3 components delivered to mothers:*Intervention*: Beverage consumption, physical activity, parental role modeling. *No control group*.	Dose: Phase 1: Four biweekly interactive group lessons delivered over 2 months. Phase 2: 2 hour lessons delivered by promotora. Six promotora led monthly group community. activities (i.e. grocery store field trips). Fidelity: Investigator supervision during group sessions but fidelity not reported.	X	Maternal BMI decreased significantly. Child BMI percentile did not decrease significantly.
Fitzgibbon et al., 2013 [55]	Head Start Pre-schools	Delivered by trained early childhood educators and facilitators in both Spanish and English	2 components delivered to children: *Intervention*: PA and Nutrition education. *Control*: General health. 3 components delivered to parents: *Intervention*: PA and Nutrition education, parenting.	Dose: *Child intervention*: 3 days/week for 40 minutes in classrooms (20 min of nutrition and 20 min of aerobic activity) for 14 wks + CD to supplement curriculum; *Parent intervention*: 6 weekly 90-minute classes + CD to reinforce materials. *Control*: Weekly newsletters about health and safety topics for 14 wks. Fidelity: Not reported.	X	Downward trend for BMI-z for both intervention and control.

X = no effect on obesity related outcomes; √= had an effect on obesity related outcomes

EFNEP = Expanded Food and Nutrition Education Program, ESL = English-as-a-Second-Language, PA = physical activity, WC = waist circumference

### Acculturation Effects

Across all interventions (adults, children, and pilot studies, N = 20), only five studies measured degree of acculturation, either through proxy measures or acculturation scales [36, 45, 46, 48, 55]. Of these, one reported that acculturation did not mediate the intervention effect during the intervention period [36]. The acculturation index used in this study, however, relied on proxy measures of acculturation. The other interventions did not state whether they tested acculturation as a possible moderator of the results [45, 46, 48, 55].

## Discussion

The changing demographics of the US population, and the prevalence of overweight and obesity among immigrants, and racial/ethnic groups in general, make it imperative to identify effective interventions to prevent and control obesity. This systematic review identified 20 relevant studies, ten completed with adults (three described as pilot studies), and ten completed with preschool aged children (five described as pilot studies). The majority of interventions targeted Latinos, who were predominately of Mexican-origin. Several of the interventions were effective at improving health outcomes, including BMI and other obesity-related measurements outcomes; however most of the study designs limit our ability to infer causality from the intervention since they did not include a control group. We identified key elements important to consider in future intervention efforts targeting obesity and identified a number of limitations in this research and areas for future direction.

### Strengths of Intervention Studies

Most of the interventions for adults addressed several behaviors, including nutrition and physical activity. Those that were successful among children also targeted multiple behaviors associated with obesity, including diet, physical activity, sleep, and screen time, in addition to parenting skills related to these behaviors. Given that the development of obesity in childhood and subsequently in adulthood involves interactions among multiple factors that shape diet and physical activity, it is important to try and target these simultaneously to affect obesity-related outcomes [56]. In addition, among children, interventions that showed stronger effects focused primarily on the caregiver and those that were pre-school or child care based did not have an effect. Targeting caregivers among immigrant populations appears to be sufficient and beneficial in obesity prevention interventions, similar to what has been observed with other populations [57]. It is also important to note that immigrants may have low levels of health literacy; therefore targeting caregivers not only improves health outcomes for children but may also improve their health literacy.

Interventions which showed positive effects for obesity in both adults and children had a cultural focus. This focus was accomplished by incorporating an engagement component and/or participatory approach to the interventions that included integrating within community structures and settings, and leveraging community resources such as bilingual workers during project implementation [58]. In general, interventions that did not have an engagement component, a community structure or a participatory approach did not work. Community-engagement strategies were found in four of the five effective adult interventions. Similarly, three of the child interventions that included a home-engagement approach that addressed parenting practices and promoted a healthy home environment were found to be effective. These findings underscore the importance of designing and implementing interventions for immigrants in a community setting and involving the community in the research process to ensure successful outcomes [59•, 60]. Taking a community-based approach, which was evident in most of the successful adult interventions, allows local groups and systems to tackle a health problem through a variety of methods that may not be known by outside researchers or public health professionals. This is important since communities present a wealth of assets ranging from human capacity, indigenous knowledge, and clearly identifiable leadership, and reasonably predictable infrastructure [61]. Furthermore, communities share proximities, problems, resources, and attributes that can be harnessed and used in positive ways [61].

### Limitations of Intervention Studies

Among adults, the studies that found an effect were predominantly quasi-experimental. Although such a design improves feasibility of implementation, it also poses many challenges to drawing causal inferences due to concerns of internal validity. Thus, it is possible that intervention effects were due to extraneous factors that were not controlled for and therefore speaks to the need for more robust study designs to determine whether the positive findings can be replicated. In addition, interventions should include long-term outcomes to determine whether positive effects can be sustained over time. For example, the adult interventions included in this review examined outcomes at the 2- to 6-month period. Similar time frames were also observed for interventions targeting children. Researchers need to consider the use of standard terminology when describing their interventions to allow for greater comparability across studies. For example, three of the interventions had a “home” based component but, Yin et al.’s [44] use of the term “home” involved reaching parents through peer-led education during child care pick up times, and did not take place in the home.

It is also worth noting that attrition rates were high (>40 %) for three of the seven adult interventions and moderately high for three of the remaining adult interventions (20-40 %). These rates are not uncommon among low-income and vulnerable populations whereby frequent moving, fluctuating employment, illness among other barriers are common [62]. Interestingly, attrition rates for interventions targeting children were much lower. It is possible that when the goal of the intervention is to improve the health of their child, caregivers are less likely to drop out. Future work should continue to seek ways to overcome these barriers and reduce attrition rates.

Finally, this review focused exclusively on BMI and other measured obesity-related outcomes. Thus, we excluded studies that only captured self-reported behavior changes. In addition, although some of the studies included in this review had outcomes that included self-reported behaviors, we did not report on these findings. Researchers are encouraged to review the original source articles for details on behavior changes achieved and the dose needed for these types of changes.

### Conclusions and Future Research Direction and Policy Implication

Although existing literature discusses the influence of acculturation on immigrant health, most interventions do not consider the possible moderating role of acculturation on obesity-related outcomes. There is overwhelming evidence that the weight gain experienced by immigrants is closely linked to their level of acculturation [63••]. Many of the interventions did not consider acculturation explicitly, which highlights an important gap in the obesity intervention literature for immigrant populations. It may also be important to target interventions to recent immigrants to the US in order to prevent weight gain associated with increased time in the US. In addition, although most research uses surrogate measures of acculturation such as length of stay or generation status, this research is complicated by two theoretical acculturation models that have dominated the field: the linear or unidirectional model (UDM) and the bi-dimensional model (BDM). The emphasis of the UDM is on assimilation, stressing that it is not possible for an individual to be a “fully integrated member of two cultures with two differing sets of cultural values” [64]. Flannery and colleagues define the UDM as “the shedding off of an old culture and the taking on of a new culture … [and] describes only one outcome of acculturation – assimilation’ (p. 1035) [65]. The UDM concept of acculturation therefore is narrow and does not permit the identification of those who are bicultural. To overcome weaknesses associated with the UDM, researchers have proposed the BDM, which takes into account two independent cultural orientations - the home and host cultures [64]. Combining these cultural orientations provide four acculturation outcomes [64, 66–68]: Traditional orientation or separation (keeping loyalty to traditional culture without recognizing the host/dominant culture); Assimilation or the ‘melting pot’ theory of acculturation (rejecting traditional culture to fully embrace the dominant culture); Integration or bicultural orientation (retaining cultural identity at the same time moving to join the dominant society); and Marginalization (rejecting traditional culture while failing to connect with the dominant culture by exclusion or withdrawal). Acculturation conceptualized as a bi-dimensional process is likely the soundest approach for future research given the effects of cultural exchange on the lifestyle of the acculturating group, including food habits and food choices, body image, physical activity patterns, and celebration [69].

It is worth noting that almost all of the interventions reviewed involved Latino populations. Although Latino populations are growing and are one of the largest in the US, other groups, such as Chinese and (Asian) Indians are also growing. Although Novotny et al. [36] targeted a different population in Hawaii, and Wieland et al. [50] included Somali and Cambodian women, no studies were identified for this review that targeted non-Latino immigrant populations. This lack of focus on non-Latino immigrant populations is ignoring other populations such as Asians who also have increased risk of developing obesity with increased duration in the US [70]. It is therefore unclear whether the general findings of our systematic review would be similar if studies included non-Latino immigrant populations. Although there are ongoing interventions involving other immigrant groups [71–73], future interventions need to include other immigrant populations in the US, specifically those that are rapidly growing [74].

## Conclusion

In summary, the number of obesity prevention interventions among immigrant populations identified in this systematic review does not reflect the rapid demographic and cultural transformation that the US has experienced over the last two decades. There is a paucity of data and little robust evidence to inform health policies and programs geared toward reducing migration-related obesity inequalities. Well-designed and culturally competent studies to evaluate the effectiveness of behavioral and lifestyle interventions in preventing obesity among immigrant populations are urgently needed. If this focus is not prioritized, there is the potential to increase the risk of a widening the gap in health status among immigrant populations.
